# Ceratitis

**Published:** 1860-10

**Authors:** 


*Ceratitis.*—In reports from St. Bartholomew Hospital, it appears that in congenital syphilitic ceratitis remarkable peculiarities are invariably found in the prominent teeth. The incisors become narrower from the gums to their cutting edges, with very much rounded corners, almost pointed, except that through the point a notch is found extending to the gum on the frontal surface, as a shallow groove. This is strongly marked in the lower incisors. In such a condition the teeth decay near the gums, and soon are lost from caries out of the mouth. B.

